# The Role of the miR-548au-3p/CA12 Axis in Tracheal Chondrogenesis in Congenital Pulmonary Airway Malformations

**DOI:** 10.1155/2023/6428579

**Published:** 2023-02-17

**Authors:** Jiahang Zeng, Xinwen Zhang, Qinglin Yang, Junzheng Peng, Fenghua Wang, Jue Tang, Yingyi Xu, Dongmei Huang, Jianhua Liang, Le Li

**Affiliations:** ^1^Department of Thoracic Surgery, Guangzhou Women and Children's Medical Center, Guangzhou Medical University, Guangzhou 510623, China; ^2^Department of Anaesthesiology, Guangzhou Women and Children's Medical Center, Guangzhou Medical University, Guangzhou, China

## Abstract

**Background:**

Literature has identified differentially expressed miRNAs in congenital pulmonary airway malformation (CPAM). However, the functional role of these miRNAs in CPAM remains unclear.

**Methods:**

We obtained diseased lung tissues as well as adjacent normal lung tissue from CPAM patients attending the centre. Hematoxylin and eosin (H&E) and Alcian blue staining were performed. Differentially expressed mRNA expression profile was CPAM tissue, and matched normal tissue specimens were examined by high-throughput RNA sequencing. CCK-8 assay, EdU staining, TUNEL staining, flow cytometry, and the Transwell assay were performed to investigate the effect of miR-548au-3p/CA12 axis on proliferation, apoptosis, and chondrogenic differentiation in rat tracheal chondrocytes. mRNA and protein expression levels were determined using reverse transcription-quantitative PCR and western blot analysis, respectively. The relationship between miR-548au-3p and CA12 was evaluated using the luciferase reporter assay.

**Results:**

The expression level of miR-548au-3p was significantly increased in diseased tissues compared with normal adjacent tissues from patients with CPAM. Our results indicate that miR-548au-3p functions as a positive regulator in rat tracheal chondrocyte proliferation and chondrogenic differentiation. At molecular level, miR-548au-3p promoted N-cadherin, MMP13, and ADAMTS4 expressions and reduced E-cadherin, aggrecan, and Col2A1 expressions. CA12 has been previously reported as a predicted target of miR-548au-3p, and here, we show that overexpression of CA12 in rat tracheal chondrocyte mimics the effects of inhibition of miR-548au-3p. On the other hand, CA12 knockdown reversed the effects of miR-548au-3p on cell proliferation, apoptosis, and chondrogenic differentiation.

**Conclusions:**

In conclusion, the miR-548au-3p/CA12 axis plays a role in the pathogenesis of CPAM and may lead to identification of new approaches for CPAM treatment.

## 1. Introduction

Congenital pulmonary airway malformation (CPAM), also named as congenital cystic adenomatoid malformation (CCAM), is a rare polycystic lesion of the lung, with an incidence rate of 1 : 11000 ~1 : 35000 in newborns [1, 2]. According to the Stocker classification, CPAM is classified into five types, namely, type 0 (3%), type I (60-70%), type II (15-20%), type III (5-10%), and type IV (10-15%) [3, 4]. Imaging and histopathological analysis of biopsies are the most commonly used tools for the clinical diagnosis for CPAM; however, CPAM can be asymptomatic, easily missed, or misdiagnosed, leading to increased risk of tumor development [5–7]. The pathogenesis of CPAM is complex, calling for a better understanding of the molecular mechanisms underlying its development and progression.

During lung development, bronchial atresia can cause excessive interstitial hyperplasia, and an imbalance between cell proliferation and apoptosis can trigger defective branching morphogenesis of the lung [8].

MicroRNAs (miRNAs), which are highly conserved short noncoding RNA molecules, play an essential role in the onset and progression of lung diseases [9, 10] by regulating a variety of biological functions, including cell differentiation and proliferation and organ morphogenesis [11, 12]. The expression and role of miRNAs in CPAM were recently reported in a recent study by Zeng et al. [13], who identified differentially expressed miRNAs in CPAMs, using miRNA chip analysis, in patients with CPAM. Specifically, the authors reported upregulation of miR-4523 and miR-548au-3p and downregulation of miR-21-3p, miR-590-3p, and miR-146a-3p in patients with type I-II CPAM compared with adjacent lesion tissue. Additional research suggested that miR-4523 overexpression attenuated dexamethasone-induced reactive oxygen species production, oxidative injury, and cell apoptosis in human osteoblasts [14]. Further, Chen et al. [15] reported that miR-21-3p may underlie the effect of cigarette smoke extract-exosomes on macrophage polarization. miR-590-3p was also identified as factor in chronic obstructive pulmonary disease based on data from the NCBI-GEO database, as reported by Li et al. [16]. In addition, miR-146a-3p knockdown decreased lipopolysaccharide-induced lung epithelial cell apoptosis and alleviated inflammation and oxidative stress in an in vivo model of acute lung injury [17]. So far, the involvement of miR-548au-3p in the pathogenesis of lung diseases or CPAM remains unclear.

miRNAs regulate posttranscriptional gene expression via binding to the 3′-untranslated region of mRNAs, which usually have hundreds of in silico predicted targets [18].

In this study, we set to investigate the role of miR-548au-3p and its downstream regulators in CPAM. Specifically, we used tracheal chondrocyte to analyze the effect of a miR-548au-3p mimic or inhibitor on apoptosis, mobility, and chondrogenic differentiation. We performed high-throughput mRNA sequencing to screen for differentially expressed targets of miR-548au-3p and potential direct downstream regulators of tracheal chondrocytes.

## 2. Materials and Methods

### 2.1. Tissue Samples

Lung tissues were harvested from patients with type I CPAM (*n* = 20) being treated at Guangzhou Women and Children's Medical Center, China, between Jan. 2020 and Dec. 2021; CPAM tissues were collected, and matched adjacent tissues were used as normal controls (*n* = 20). Signed informed consent for all patients was obtained prior to tissue collection. This study was approved by the Ethics Committee of Guangzhou Women and Children's Medical Center, China.

### 2.2. Histological Analysis

Histological analysis was done using hematoxylin and eosin (H&E) and Alcian blue staining. Tissue specimens were fixed, dehydrated, and then embedded in paraffin and cut into 5 *μ*m sections. Sections were then deparaffinized, rehydrated, and stained with1% Alcian blue (Sigma-Aldrich) for 30 min for detection of proteoglycan and mucopolysaccharides or with H&E staining.

For Alcian blue staining of cells, rat tracheal chondrocytes were washed three times, treated with 0.1 mol/l hydrochloric acid solution, and incubated in a solution of 1% Alcian blue staining overnight.

### 2.3. Cell Culture

Rat tracheal chondrocytes were purchased from Procell Life Science & Technology Co., Ltd. (Wuhan, China) and grown in DMEM (Thermo Fisher Scientific) with 10% FBS in an incubator (37°C and 5% CO_2_).

### 2.4. High-Throughput mRNA Sequencing Analysis

Extraction of total RNA from tissues was performed with TRIzol reagent (company), and RNA quality was determined using the Agilent 2100 Bioanalyzer (Agilent Technologies, Santa Clara, CA, USA). RNA was quantified and amplified with GeneChip 3′IVT Express Kit (Affymetrix, CA, USA). After purification by RNAClean XP Kit (Cat. A63987, Beckman Coulter, Inc., Kraemer Boulevard Brea, CA, USA), amplified RNA was fragmented and processed using the GeneChip Hybridization Wash and Stain Kit (Affymetrix). Raw data of dyed chips were scanned using a GeneChip Scanner 3000 (Affymetrix). Differentially expressed genes (DEGs) were identified between CPAM samples and normal samples by using the Limma package (provide info) under the criterion of false discovery rate (FDR) < 0.05 and |log_2_ fold change (FC)| > 1.

### 2.5. Cell Transfection

The miR-548au-3p mimics, inhibitor and negative control (NC), CA12 overexpression plasmid, and small interfering RNA (siRNA) targeting CA12 and siRNA NC were provided by Guangzhou RiboBio Co., Ltd. and transfected into rat tracheal chondrocytes. In rescue experiments, rat tracheal chondrocytes were cotransfected with miR-548au-3p inhibitor and CA12 siRNA. All transfections were carried out for 48 h using Lipofectamine 2000 (Invitrogen, Carlsbad, CA, USA), in accordance to manufacturer's instructions.

### 2.6. Reverse Transcription-Quantitative PCR

Following RNA extraction using TRIzol (Invitrogen, USA), complementary DNA (cDNA) with cDNA was synthesized using a Reverse Transcription Kit (Thermo Fisher Scientific). We performed reverse transcription-quantitative PCR using the Power SYBR® Green Master Mix (Thermo Fisher Scientific), on an ABI 7500 Fast Real-Time PCR instrument (Applied Biosystems, Foster City, CA, USA). The primer sequences used are listed in Table 1. We calculated relative miRNA and mRNA levels using the 2^−*ΔΔ*CT^ method.

### 2.7. CCK-8 Assay

Cell viability of rat tracheal chondrocytes was determined using a CCK-8 kit (Dojindo, Kumamoto, Japan), according to the manufacturer's instructions. Approximately 3 × 10^3^ transfected cells were seeded into each well of a 96-well plate and cultured for 24, 48, 72, and 96 h. At each time point, cells were incubated with 10 *μ*l CCK-8 solution for 2 h at 37°C, and optical densities (OD) were measured at 450 nm.

### 2.8. Cell Proliferation Assay

Cell proliferation of rat tracheal chondrocytes was assessed by EdU staining. In brief, cells were seeded in 24-well plates and the Cell-Light EdU DNA Cell Proliferation Kit (Guangzhou RiboBio Co., Ltd.) was used, according to the manufacturer's instructions.

### 2.9. Terminal Transferase-Mediated DNA End Labelling (TUNEL) Assay

The TUNEL assay was used to examine the presence of fragmented DNA in apoptotic cells. After trypsinization, cells were resuspended in fresh media, washed with PBS, fixed with 4% formaldehyde for 25 min at 4°C, and permeabilized with 0.2% Triton X-100 for 2 min on ice. Next, cells were washed again with PBS and labeled for 60 min with 50 *μ*l of TUNEL test solution in darkness at 37°C. Cells were then incubated with DAPI reagent and visualized under a fluorescence microscopy.

### 2.10. Flow Cytometry

Cell apoptosis of rat tracheal chondrocytes was determined using the FITC Annexin V Apoptosis Detection Kit (BD Pharmingen, Franklin Lakes, NJ, USA) according to the manufacturer's instructions. In brief, cells were trypsinized, centrifuged at 1500 rpm for 5 min, and resuspended in 1x binding buffer. Cells were then stained for 5 min in the dark with 5 *μ*l of FITC Annexin V and propidium iodide. Next, the numbers and percentage of apoptotic cells were calculated using a cell sorter (BD FACSAria Cell Sorter, Chestnut Hill, MA).

### 2.11. Transwell Assay

To examine cell migration, rat tracheal chondrocytes (5 × 10^4^) were resuspended in 100 *μ*l serum-free medium and seeded into the upper 24 wells of a Transwell chamber with 8 *μ*m pore size (Corning, Inc.). In the lower chambers, we added 500 *μ*l of DMEM containing 20% FBS as a chemoattractant. After incubation for 24 h at 37°C, the cells that had migrated into the lower chambers were fixed with 4% paraformaldehyde for 15 min and stained with 0.5% crystal violet both at room temperature for 30 min. Migrated cells in five randomly fields of view were photographed and counted using an inverted microscope.

### 2.12. Luciferase Reporter Assay

The luciferase reporter assay was used to validate the relationship between miR-548au-3p and CA12, predicted based on analysis of putative miR-548au-3p binding site in CA12 by TargetScan. In brief, the binding sites of putative wild-type (WT) and mutant (MUT) miR-548au-3p in the 3′-UTR of CA12 were inserted into a pmirGLO-Report luciferase vector (Promega, Madison, WI, USA) to generate corresponding CA12-WT and CA12-MUT plasmids. HEK-293 cells were cotransfected with CA12-WT or CA12-MUT, together with miR-548au-3p mimics or NC, using Lipofectamine 2000 (Invitrogen, city, country) for 48 h. The relative luciferase activity was analyzed with the luciferase reporter assay system (Promega, Madison, WI, USA).

### 2.13. Western Blot Analysis

Total protein was extracted using the RIPA lysis buffer kit (Beyotime, Shanghai, China), and total protein was mixed with 1x loading buffer. 10% SDS-PAGE gel electrophoresis was performed, and samples were transferred onto PVDF membranes (Millipore Corp. Billerica, MA, USA). Then, membranes were incubated with 5% skim milk and incubated with primary antibodies overnight at 4°C. Membranes were then incubated with secondary peroxidase-conjugated antibodies for 1 h at room temperature. Finally, a SuperSignal chemiluminescent substrate (Millipore, Billerica, MA, USA) was used to visualize protein bands. Primary antibodies for this study were specific for CA12 (Boster, Cat. No. A04063, 1 : 1000), LONRF3 (GeneTex, Cat. No. GTX112150, 1 : 2000), MAP2 (Boster, Cat. No. A01201, 1 : 2000), THBS1 (Boster, Cat. No. PB0471, 1 : 2000), PPID, E-cadherin (Beyotime, Cat. No. AF6759, 1 : 1000), N-cadherin (Beyotime, Cat. No. AF5237, 1 : 800), aggrecan (Abcam, Cat. No. ab3778, 1 : 1000), Col2A1 (Boster, Cat. No. A00517, 1 : 2000), MMP13 (Proteintech, Cat. No. 18165-1-AP, 1 : 3000), ADAMTS4 (Proteintech, Cat. No. 11865-1-AP, 1 : 600), and GAPDH (Abcam, Cat. No. ab9485, 1 : 2000). GAPDH was used as internal control.

### 2.14. Statistical Analysis

GraphPad Prism 8.0 was used to analyze the data. Data were expressed as mean ± standard derivation from three independent experiments. Gene expression levels between CPAM samples and control samples were compared using an independent *t*-test. Analysis for multiple groups was conducted using one-way ANOVA followed by Dunnett's test. A *p* value < 0.05 was deemed as statistically significant.

## 3. Results

### 3.1. miR-548au-3p Is a Potential Target in CPAM Pathogenesis

**Figure 1:**
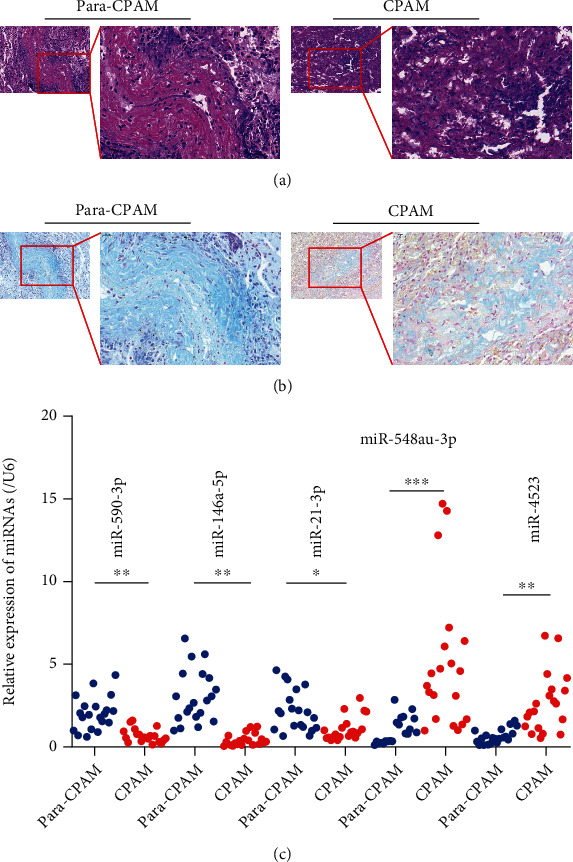
miR-548au-3p is a potential target involved in pathogenesis of CPAM. Representative scans of (a) hematoxylin and eosin (H&E) and (b) Alcian blue staining in lung tissues from CPAM patients. (c) Expression of miR-590-3p, miR-146a-5p and miR-21-3p, miR-548au-3p, and miR-4523 was determined in lung tissues from CPAM patients (*n* = 20) and adjacent tissues (*n* = 20).

We determined the expression levels of previously identified miRNA markers, in CPAM using lung tissues from type I/II CPAM patients. Inspection of H&E staining indicated more multiple cysts with different sizes in type I/II CPAM-derived lung tissues compared with adjacent normal tissue (Figure 1(a)). As shown in Figure 1(b), Alcian blue staining revealed excess mucous and goblet cell hyperplasia in diseased tissues from patients with type I/II CPAM, in contrast with normal tissue. We used reverse transcription-quantitative PCR to measure several identified miRNA markers. As shown in Figure 1(c), a significant decrease in the expression levels of miR-590-3p, miR-146a-5p, and miR-21-3p was observed in diseased tissues from CPAM patients, compared with normal adjacent tissue. In addition, diseased tissues were characterized by increased expression of miR-548au-3p and miR-4523. Given that miR-548au-3p expression level exhibited the greatest increase among detected five miRNAs, this miRNA was selected for further analyses.

### 3.2. miR-548au-3p Contributed to Rat Tracheal Chondrocyte Cell Proliferation and Chondrogenic Differentiation

**Figure 2:**
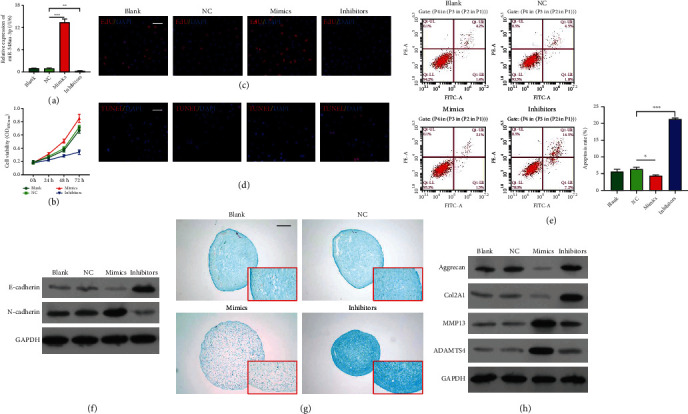
miR-548au-3p promoted rat tracheal chondrocyte proliferation and differentiation. Rat tracheal chondrocytes were transfected with miR-548au-3p mimics, inhibitor, or negative control (NC). (a) Transfection efficiency assessed by PCR. (b) Cell viability of rat tracheal chondrocytes assessed by CCK-8 assay. (c) Cell proliferation ability assessed by EdU staining. Red fluorescence: EdU; blue fluorescence: DAPI (scale bar = 50 *μ*m). (d) Apoptosis levels assessed by TUNEL assay (scale bar = 50 *μ*m). Red fluorescence: TUNEL; blue fluorescence: DAPI. (e) Cell apoptosis determined by flow cytometry with Annexin V/propidium iodide staining. (f) Protein levels of E-cadherin and N-cadherin. (g) The effect of miR-548au-3p on differentiation of rat tracheal chondrocytes assessed using Alcian blue staining (scale bar = 100 *μ*m). (h) Aggrecan, Col2A1, MMP13, and ADAMTS4 protein levels assessed by western blot. ^∗^*p* < 0.05, ^∗∗^*p* < 0.01, and ^∗∗∗^*p* < 0.001, compared with NC.

To determine whether miR-548au-3p was involved in CPAM *in vitro*, miR-548au-3p expression was altered in rat tracheal chondrocytes by transfection with miR-548au-3p mimics or inhibitor (Figure 2(a)). The results from CCK-8 assay (Figure 2(b)) and EdU staining (Figure 2(c)) consistently indicated that viability and proliferation rate of rat tracheal chondrocytes were significantly elevated after miR-548au-3p overexpression but were reduced after miR-548au-3p knockdown. TUNEL-positive cells were reduced after miR-548au-3p overexpression but were elevated after miR-548au-3p knockdown (Figure 2(d)). Similar results were obtained using flow cytometry (Figure 2(e)). These results are in agreement with changes of E-cadherin and N-cadherin protein levels, as assessed by western blot (Figure 2(f)). Transwell experiments revealed positive effects of miR-548au-3p on cell migration (Figure S1). Alcian blue staining suggested that miR-548au-3p promoted expression of glycosaminoglycans (Figure 2(g)). Furthermore, we analyzed the expressions of chondrogenic differentiation-related factors aggrecan, Col2A1, MMP13, and ADAMTS4 by western blot analysis. As depicted in Figure 2(h), overexpression of miR-548au-3p in rat tracheal chondrocytes promoted expression of MMP13 and ADAMTS4 but reduced expression of aggrecan and Col2A1. Knockdown of miR-548au-3p led to opposite results.

### 3.3. Identification of DEGs Involved in CPAM Pathogenesis

**Figure 3:**
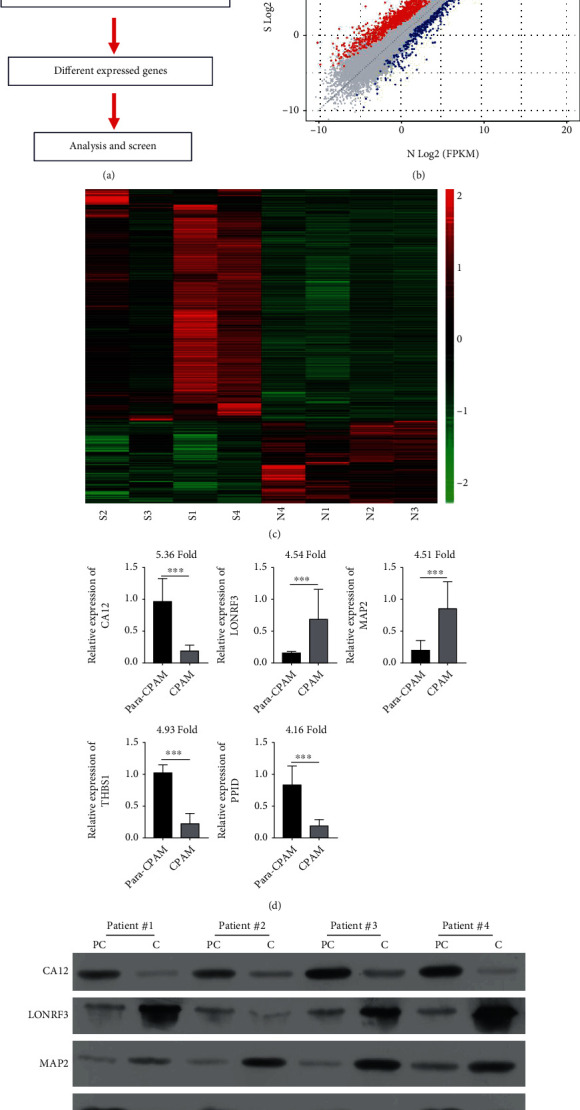
Identification of DEGs involved in the pathogenesis of CPAM. (a) Flowchart of the study design and samples at each stage of analysis. (b) Scatterplot of mRNA expression variation between diseased CPAM and normal tissues. (c) Hierarchical cluster of gene expression profiles from microarray assays. Expression of CA12, LONRF3, MAP2, THBS1, and PPID at mRNA (d) and protein (e) levels in lung tissues from CPAM and adjacent normal tissues. ^∗∗∗^*p* < 0.001, compared with normal.

We investigated the role of miR-548au-3p and its downstream signaling pathways in the pathogenesis of CPAM using high-throughput mRNA sequencing analysis. The study design and the samples included at every stage of the analysis are described in Figure 3(a). The differential mRNA expression between CPAM and normal tissues is conveyed in the scatter plot of gene expression profile (Figure 3(b)). After further screening, the top 8 DEGs included 5 downregulated and 3 upregulated genes, as described by a hierarchical cluster (Figure 3(c)). We further confirmed downregulation of CA12, THBS1, and PPID and the upregulation of LONRF3 and MAP2 in lung tissues derived from CPAM patients compared with controls at mRNA (Figure 3(d)) and protein levels (Figure 3(e)).

### 3.4. CA12 As a Direct Target of miR-548au-3p

**Figure 4:**
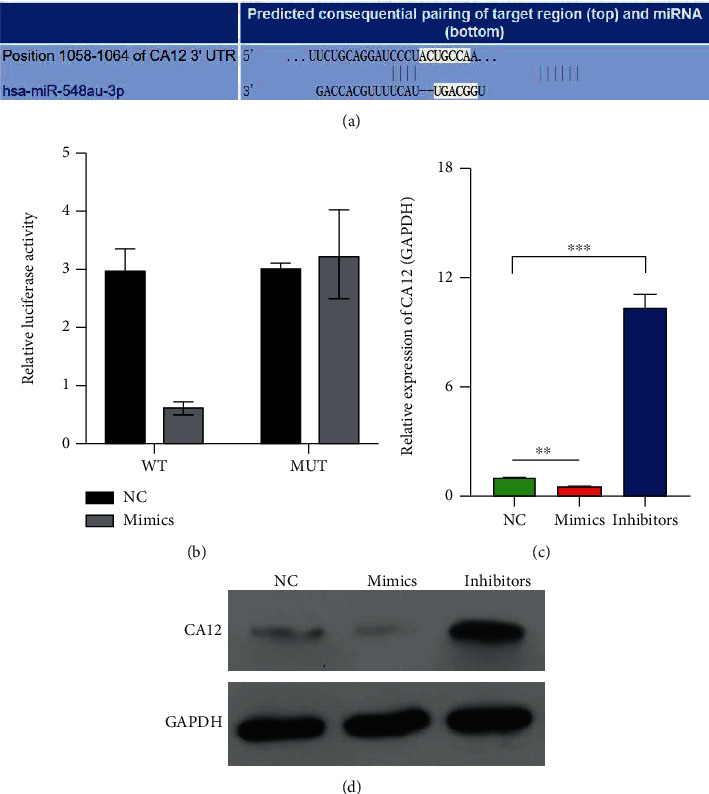
CA12 as a direct target of miR-548au-3p. (a) Binding sites between miR-548au-3p and the 3′-UTR region of CA12 mRNA. (b) HEK-293 cells were cotransfected with miR-548au-3p mimics or NC and wild-type or mutant CA12 3′-UTR, followed by luciferase reporter assay. CA12 mRNA (c) and protein (d) levels in rat tracheal chondrocytes after transfection with miR-548au-3p mimics or inhibitor. ^∗∗^*p* < 0.01 and ^∗∗∗^*p* < 0.001, compared with NC.

Among the identified five genes by high-throughput mRNA sequencing analysis, based on data from the TargetScan database, we hypothesized that CA132 was a direct target of miR-548au-3p via, as shown in Figure 4(a). Results using a luciferase reporter showed a significant decrease on the luciferase activity of the CA12 WT plasmid after miR-548au-3p mimic transfection in in rat tracheal chondrocytes, compared with transfection with a negative control. The luciferase activity of CA12 MUT plasmid was not significantly changed (Figure 4(b)). Moreover, the expression of CA12 mRNA (Figure 4(c)) and protein (Figure 4(d)) decreased after transfection with miR-548au-3p and increased after transfection with a miR-548au-3p inhibitor.

### 3.5. CA12 Suppressed Rat Tracheal Chondrocyte Proliferation and Differentiation

**Figure 5:**
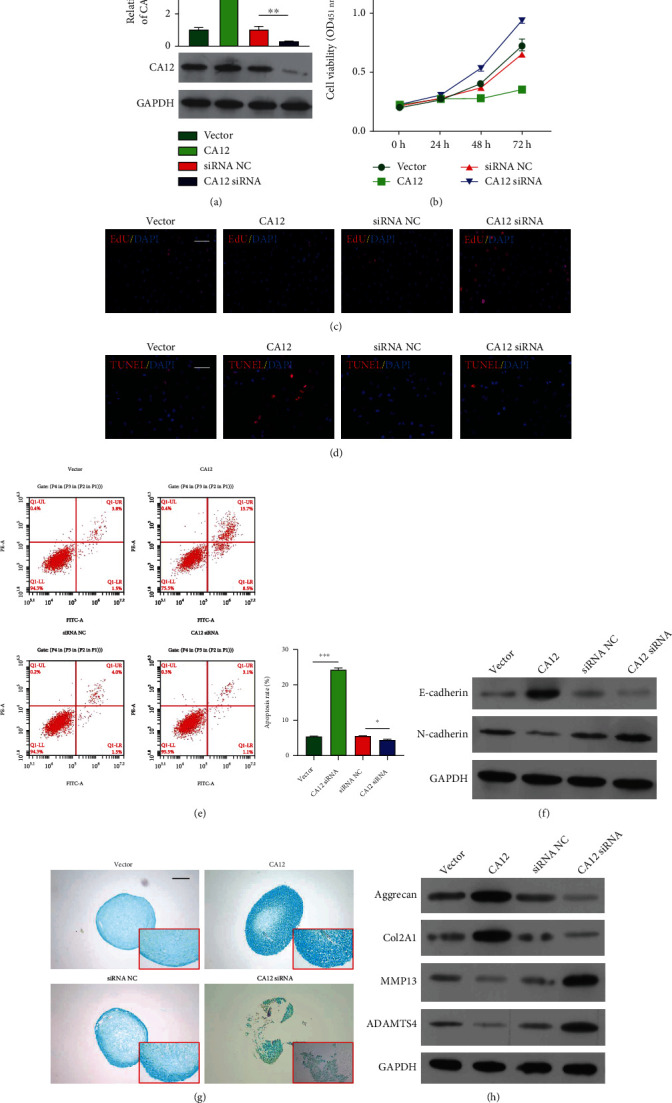
CA12 suppressed rat tracheal chondrocyte proliferation and differentiation. Rat tracheal chondrocytes were transfected with CA12 overexpression plasmid or CA12 siRNA. (a) Transfection efficiency was determined by western blot. (b) Viability of rat tracheal chondrocytes assessed by CCK-8 assay. (c) Cell proliferation ability assessed by EdU staining. Red fluorescence: EdU; blue fluorescence: DAPI (scale bar = 50 *μ*m). (d) Cell apoptosis levels assessed by TUNEL staining (scale bar = 50 *μ*m). Red fluorescence: TUNEL; blue fluorescence: DAPI. (e) Cell apoptosis assessed by flow cytometry with Annexin V/propidium iodide staining. (f) Protein expression of E-cadherin and N-cadherin. (g) The effect of miR-548au-3p on differentiation of rat tracheal chondrocytes assessed with Alcian blue staining (scale bar = 100 *μ*m). (h) Aggrecan, Col2A1, MMP13, and ADAMTS4 protein levels detected by western blot. ^∗^*p* < 0.05, ^∗∗^*p* < 0.01, and ^∗∗∗^*p* < 0.001, compared with vector or siRNA NC.

To study the functional role of CA12, rat tracheal chondrocytes were transfected with a CA12 overexpression plasmid or a CA12 siRNA as negative control. CA12 overexpression and knockdown were confirmed by western blot (Figure 5(a)). Subsequent experiments showed that overexpression of CA12 suppressed cell viability (Figure 5(b)) and proliferation (Figure 5(c)) and promoted apoptosis (Figures 5(d) and 5(e)) of rat tracheal chondrocytes. The expression of E-cadherin increased and N-cadherin decreased after CA12 overexpressed (Figure 5(f)). In addition, overexpression of CA12 suppressed cell migration (Figure S2). Similar to results obtained by miR-548au-3p knockdown, overexpression of CA12 inhibited the expression of glycosaminoglycans (Figure 5(g)) and downregulated expression of MMP13 and ADAMTS4 expressions and upregulated expression of aggrecan and Col2A1 (Figure 5(h)).

### 3.6. CA12 Knockdown Reversed miR-548au-3p Inhibitor-Mediated Suppressive Effects on Rat Tracheal Chondrocytes

**Figure 6:**
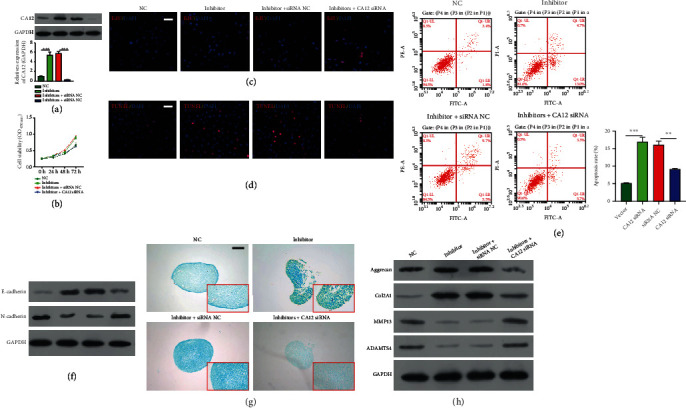
Knockdown of CA12 abolished miR-548au-3p inhibitor mediated effects on rat tracheal chondrocytes. Rat tracheal chondrocytes were cotransfected with CA12 siRNA and miR-548au-3p inhibitor for 48 h. (a) Transfection efficiency was determined by western blot. (b) Viability of rat tracheal chondrocytes assessed by CCK-8 assay. (c) Cell proliferation ability assessed by EdU staining. Red fluorescence: EdU; blue fluorescence: DAPI (scale bar = 50 *μ*m). (d) Cell apoptosis assessed by TUNEL staining (scale bar = 50 *μ*m). Red fluorescence: TUNEL; blue fluorescence: DAPI. (e) Cell apoptosis assessed by flow cytometry with Annexin V/propidium iodide staining. (f) E-cadherin and N-cadherin protein levels assessed by western blot. (g) The effect of miR-548au-3p on chondrogenic differentiation of rat tracheal chondrocytes assessed by Alcian blue staining (scale bar = 100 *μ*m). (h) Aggrecan, Col2A1, MMP13, and ADAMTS4 protein levels assessed by western blot. ^∗∗^*p* < 0.01 and ^∗∗∗^*p* < 0.001, compared with NC or inhibitor siRNA NC.

To further confirm whether CA12 was involved in miR-548au-3p-mediated processes, we performed rescue experiments by cotransfecting rat tracheal chondrocytes with miR-548au-3p inhibitor and CA12 siRNA. Western blot analysis confirmed that transfection with miR-548au-3p inhibitor caused elevation of CA12, which was obviously attenuated after cotransfection with miR-548au-3p inhibitor and CA12 siRNA (Figure 6(a)). Results from the CCK-8 assay (Figure 6(b)), EdU staining (Figure 6(c)), TUNEL staining (Figure 6(d)), and flow cytometry (Figure 6(e)) indicated decrease of cell viability and proliferation, following miR-548au-3p knockdown, as well as reduction of apoptosis after CA12 knockdown. In addition, knockdown of CA12 reversed miR-548au-3p knockdown-mediated effects on cell migration (Figure S3) and alterations in EMT markers (E-cadherin and N-cadherin) (Figure 6(f)). Moreover, miR-548au-3p knockdown suppressed the expression of glycosaminoglycans (Figure 6(g)); upregulation of aggrecan/Col2A1 and downregulation of MMP13/ADAMTS4 (Figure 6(h)) were inhibited by cotransfection with miR-548au-3p inhibitor and CA12 siRNA.

## 4. Discussion

In this study, we set out to explore the role of miRNAs in the pathology of CPAM, a congenital disorder of the lung. Zeng et al. [13] previously provided genomic insights into this condition and revealed that diseased lung tissues from CPAM patients exhibited downregulation of miR-590-3p, miR-146a-5p, and miR-21-3p and upregulation of miR-548au-3p and miR-4523 expressions, in comparison to adjacent normal lung tissues. Here, we show that miR-548au-3p promoted proliferation and differentiation of rat tracheal chondrocytes. CPAM, as a developmental disorder, may occur in conjunction with other congenital anomalies which are risk factors for disease progression later in life [19]. We hypothesized that upregulation of miR-548au-3p might trigger CPAM by governing airway formation during gestation via regulation postnatal cell growth and differentiation [20]. To test this hypothesis, we use rat tracheal chondrocytes as a model, as they have been widely used to study congenital disorder-related diseases, including tracheobronchomalacia [21], cartilage regeneration [22], and lung agenesis [23]. At molecular level, we found that miR-548au-3p promoted expression of N-cadherin, MMP13, and ADAMTS4 but suppressed expression levels of E-cadherin, aggrecan, and Col2A1. Tracheal epithelial cells function as a key regulator in the defense of respiratory tract [24]. E-cadherin plays a key role in adherent junctions and hence regulates epithelial barrier function [25]. Cao et al. [26] showed that cleavage of E-cadherin disrupted airway epithelial cell barriers and suppressed cell proliferation in the respiratory mucosal surface. Secreted metalloprotease members from the ADAMTS family also play crucial roles in modulating the extracellular matrix (ECM) [27]. Previous studies reported that proliferative chondrocytes could produce cartilage extracellular matrix, including type II collagen and aggrecan, along with generation of type X collagen (COL10) and matrix metalloproteinase 13 (MMP13) [28]. Given the association between excessive interstitial hyperplasia and CPAM, we hypothesized that miR-548au-3p promoted CPAM development by regulating chondrocyte proliferation, mobility, and differentiation via regulating ECM and EMT processes.

Through high-throughput mRNA sequencing analysis, we identified the top five differentially expressed mRNAs (CA12, LONRF3, MAP2, THBS1, and PPID) in diseased CPAM versus normal lung tissue. We confirmed CA12 as a direct target of miR-548au-3p. Our functional experiments indicated that miR-548au-3p regulated cell proliferation, mobility, and differentiation in rat tracheal chondrocytes by targeting CA12. As reported by Han et al. [29], human hypoxia-treated ligamentum flavum-derived stem cells (LFSCs) expressed higher levels of aggrecan and CA12 promoted the proliferation and differentiation of these cells toward nucleus pulposus-like cells. Furthermore, as a transmembrane protein, carbonic anhydrase 12 (CA12) participates in the regulation of cellular pH in metabolically active cells/tissues via catalysis of carbon dioxide hydration and dehydration reversible reactions [30]. Kim et al. [31] suggested a role of CA12 in host defense against inhaled pathogens in the airway mucosal microenvironment and also reported cystic fibrosis-like airway infections in people with CA12 mutations.

In this work, we suggest that CA12 plays an important role in airway microenvironment and is a key regulator of the miR-548au-3p axis in the development of CPAM, although a direct relationship cannot be assumed based on current evidence.

In summary, we identified five key mRNAs in the pathogenesis of CPAM using high-throughput mRNA sequencing analysis. Moreover, we demonstrated that miR-548au-3p promoted proliferation and chondrogenesis in rat tracheal chondrocytes by targeting CA12. More in-depth studies are necessary, but, in the meantime, our present findings provide new insights into the molecular mechanisms underlying CPAM.

## Figures and Tables

**Table 1:** Primers for real-time PCR.

Name of genes	Sequences (5′→3′)
GAPDH F	TGTTCGTCATGGGTGTGAAC
GAPDH R	ATGGCATGGACTGTGGTCAT
CA12 F	AGGCCAGGAAGCATTCGTC
CA12 R	GGGAAGGGTCGTCCATGTG
U6 F	CTCGCTTCGGCAGCACA
U6 R	AACGCTTCACGAATTTGCGT
All R	CTCAACTGGTGTCGTGGA
miR-146a-5p RT	CTCAACTGGTGTCGTGGAGTCGGCAATTCAGTTGAGAACCCATG
miR-146a-5p F	ACACTCCAGCTGGGTGAGAACTGAATTCCA
miR-590-3p RT	CTCAACTGGTGTCGTGGAGTCGGCAATTCAGTTGAGACTAGCTT
miR-590-3p F	ACACTCCAGCTGGGTAATTTTATGTATAA
miR-21-3p RT	CTCAACTGGTGTCGTGGAGTCGGCAATTCAGTTGAGACAGCCCA
miR-21-3p F	ACACTCCAGCTGGGCAACACCAGTCGATG
miR-548au-3p RT	CTCAACTGGTGTCGTGGAGTCGGCAATTCAGTTGAGCTGGTGCA
miR-548au-3p F	ACACTCCAGCTGGGTGGCAGTTACTTTTG
miR-4523 RT	CTCAACTGGTGTCGTGGAGTCGGCAATTCAGTTGAGACAGCCGA
miR-4523 F	ACACTCCAGCTGGGGACCGAGAGGGCCTC

F: forward primer; R: reverse primers; RT: reverse transcription.

## Data Availability

The data used to support the findings of this study are included within the article.

## Supplementary Materials

Supplementary Materials Figure S1: cell migration was investigated after cells treated with miR-548au-3p mimics or inhibitors using Transwell assay (scale bar = 50 *μ*m). Figure S2: cell migration was investigated in CA12-overexpressed or CA12-knocked down cells using Transwell assay (scale bar = 50 *μ*m). Figure S3: cell migration was investigated after CA12 knocked down in miR-548au-3p inhibited cells (scale bar = 50 *μ*m).
