# Supplementary material for: The Role of the miR-548au-3p/CA12 Axis in Tracheal Chondrogenesis in Congenital Pulmonary Airway Malformations
Source: Oxid Med Cell Longev. 2023 Feb 17;2023:6428579. doi: 10.1155/2023/6428579 (PMC9957630; doi:10.1155/2023/6428579)
Supplement: Supplementary Materials — Figure S1: cell migration was investigated after cells treated with miR-548au-3p mimics or inhibitors using Transwell assay (scale bar = 50 μm). Figure S2: cell migration was investigated in CA12-overexpressed or CA12-knocked down cells using Transwell assay (scale bar = 50 μm). Figure S3: cell migration was investigated after CA12 knocked down in miR-548au-3p inhibited cells (scale bar = 50 μm). [file 6428579.f1.docx]

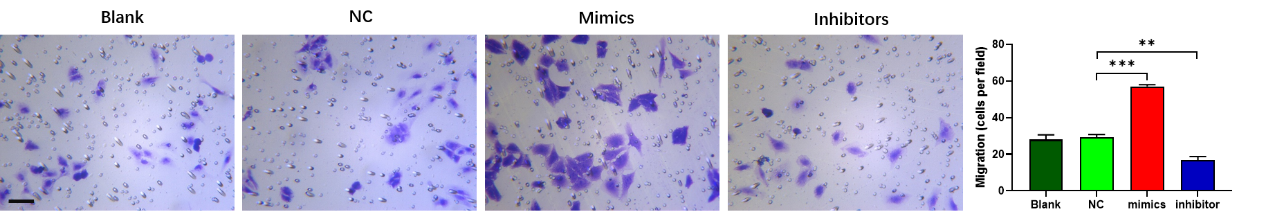
Figure S1 Cell migration was investigated after cells treated with miR-548au-3p mimics or inhibitors using Transwell assay (Scale bar =50 μm).


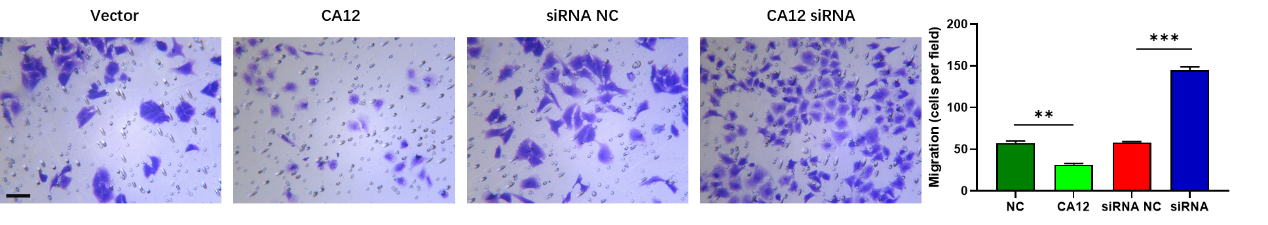
Figure S2 Cell migration was investigated in CA12-overexpressed or CA12-koncked down cells using Transwell assay (Scale bar =50 μm).


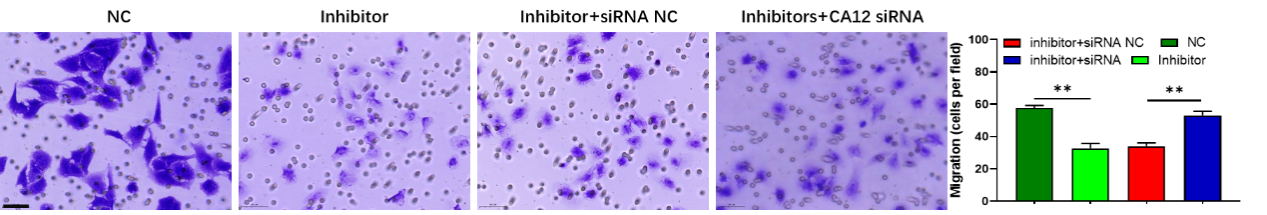
Figure S3 Cell migration was investigated after CA12 knocked down in miR-548au-3p inhibited cells (Scale bar =50 μm).
